# Letter to the Editor: Enhancing methodological rigor and clinical applicability in vancomycin powder use for infection prevention in primary joint arthroplasty

**DOI:** 10.1097/JS9.0000000000002897

**Published:** 2025-07-03

**Authors:** Kun Zhang, Fan Yang

**Affiliations:** Department of Trauma Orthopedics, Shenzhen Longhua District People’s Hospital, Shenzhen, Guangdong, China

*Dear Editor,*

We have read with great interest the article titled “*Intrawound vancomycin powder for prevention of surgical site infections in primary joint arthroplasty: an umbrella review of systematic reviews and meta-analyses*” published in *International Journal of Surgery*^[1]^. We commend the authors for their significant contribution to the ongoing efforts in infection prevention in joint arthroplasty. This umbrella review presents a comprehensive analysis of multiple systematic reviews and meta-analyses, shedding light on the potential of intraoperative vancomycin powder to reduce surgical site infections (SSI) in primary joint replacement procedures. However, after careful examination of the study, we believe there are several areas where the research can be strengthened, both in its methodological approach and clinical applicability. In line with the TITAN 2025 guideline on AI transparency ^[2]^, a generative AI-based language model was used solely for language polishing and had no bearing on the scientific content.

First and foremost, while the review incorporates a broad range of studies, it is important to note that most of these studies are retrospective and observational in nature. These types of studies inherently limit the ability to establish definitive cause-and-effect relationships. Despite the general agreement on the efficacy of vancomycin powder in reducing SSIs, the lack of randomized controlled trials (RCTs) in the included studies represents a notable limitation. We recommend that future studies employ RCTs to control for biases and more effectively measure the causal effects of vancomycin powder on infection rates.

Secondly, the data analysis in the review does not fully address the potential variations in infection rates based on the type of implant used. Although the review shows that vancomycin powder significantly reduces infection rates in general, there is a need to differentiate the outcomes based on the type of implant used, particularly between cemented and cementless prostheses. As suggested by Matziolis *et al*, the effectiveness of vancomycin powder appears to be more pronounced in cementless hip and knee arthroplasties^[3]^. A more detailed analysis of this factor could provide a more nuanced understanding of the intervention’s effectiveness across various implant types.

Furthermore, while the authors acknowledge the positive effects of vancomycin powder, they did not fully explore how different patient populations—such as elderly individuals or those who are immunocompromised—may respond to this intervention. Research has demonstrated that these populations are at a significantly higher risk for postoperative infections^[4]^. It is essential to investigate whether vancomycin powder can provide similar levels of efficacy in these vulnerable groups. Future studies should focus on assessing the specific impact of vancomycin powder on such populations, considering factors such as age, comorbidities, and immune status.

In addition to the methodological suggestions, the review did not explore the optimal dosage and timing for vancomycin powder application. Current evidence does not provide conclusive data on whether the dosage or timing of administration influences outcomes^[5]^. We strongly recommend that future research examine these variables in more depth, with a focus on identifying the most effective regimen for clinical application.

Finally, while the review primarily focuses on short-term infection rates, it does not address the long-term effects of vancomycin powder in preventing recurrent infections. Although the powder has shown efficacy in the short term, its role in preventing recurrent infections over a longer period remains unclear^[6]^. It would be beneficial for future studies to incorporate extended follow-up periods, ideally at least 12 months, to better understand the long-term effectiveness of this intervention.

In conclusion, while the review makes a significant contribution to our understanding of vancomycin powder in preventing SSIs in primary joint arthroplasty, we believe there are several areas where the study could be improved. Future research should focus on RCTs, consider variations between implant types, and explore the effects of this intervention in different patient populations. Additionally, examining dosage, timing, and long-term outcomes will be essential to fully evaluate the clinical utility of vancomycin powder in arthroplasty surgeries.

## Data Availability

No new data were generated or analyzed.

## AI assistance

A generative AI-based language model was employed solely for grammar and style editing.

## Technical comments

Correspondence: A letter to the Editor that raises issues of importance to clinical surgery and is in regard to an article recently published in the journal. The manuscript refers to an article published in a different scientific journal.
